# A 34-Year-Old Man With a Neck Mass

**DOI:** 10.1016/j.chest.2021.09.026

**Published:** 2022-03-04

**Authors:** Tamara Phiri, Joep van Oosterhout, Samuel Kampondeni, Theresa Allain, Henry C. Mwandumba

**Affiliations:** a Department of Medicine, University of Malawi College of Medicine, Blantyre, Malawi; b Malawi-Liverpool-Wellcome Trust Clinical Research Programme, University of Malawi College of Medicine, Blantyre, Malawi; c Department of Clinical Sciences, Liverpool School of Tropical Medicine, Liverpool, England

## Abstract

A 34-year-old man presented to Queen Elizabeth Central Hospital in Blantyre, Malawi with multiple enlarged right cervical lymph nodes. He had no associated constitutional symptoms. Fine-needle aspirate (FNA) of one of the lymph nodes was negative for acid-fast bacilli (AFB) by smear microscopy. The FNA specimen was not sent for histological examination. Mycobacterial culture and Xpert MTB/RIF were not available at the time. He tested positive for HIV but CD4 T-cell count was not requested at the time of HIV diagnosis, and he did not start antiretroviral therapy (ART) pending confirmation of the cause of lymphadenopathy. Excision biopsy of the lymph nodes was planned; however, the patient was lost to follow-up before the procedure was performed.

Six months after the initial presentation, the patient returned with a productive cough and constitutional symptoms. Sputum smear microscopy was positive for AFB. He started treatment for pulmonary TB, comprising rifampicin, isoniazid, pyrazinamide, and ethambutol.

Two months into TB treatment, he returned to the hospital with right-sided neck pain and a progressively enlarging right-sided neck mass.

## Physical Examination

On assessment, he was alert, with a BP of 116/70 mm Hg, pulse rate of 67 beats/min, respiratory rate of 15 breaths/min, and was afebrile. He had no finger clubbing, cyanosis, pallor, oral candidiasis, or Kaposi’s sarcoma lesions, and did not have stridor or hoarse voice. He had a large pulsatile right-sided neck mass measuring 6 cm × 6 cm. The JVP was not elevated. The apex beat was not displaced, and his heart and breath sounds were normal. The abdominal and neurological examination was normal.

## Laboratory and Radiology Findings

Hemoglobin was 13.6 g/dL, WBC count 5,400/μL, and platelets 320,000/μL, and CD4 count was 594 cells/μL. Creatinine was 1.0 mg/dL. Serum venereal disease research laboratory was negative. Blood culture and erythrocyte sedimentation rate, though the services were available at the hospital, were not requested. Additional blood tests such as C-reactive protein, lactate dehydrogenase, uric acid, and Epstein Barr virus viral load were not available, and the patient was unable to pay for these tests to be performed elsewhere.

**Figure 1:**
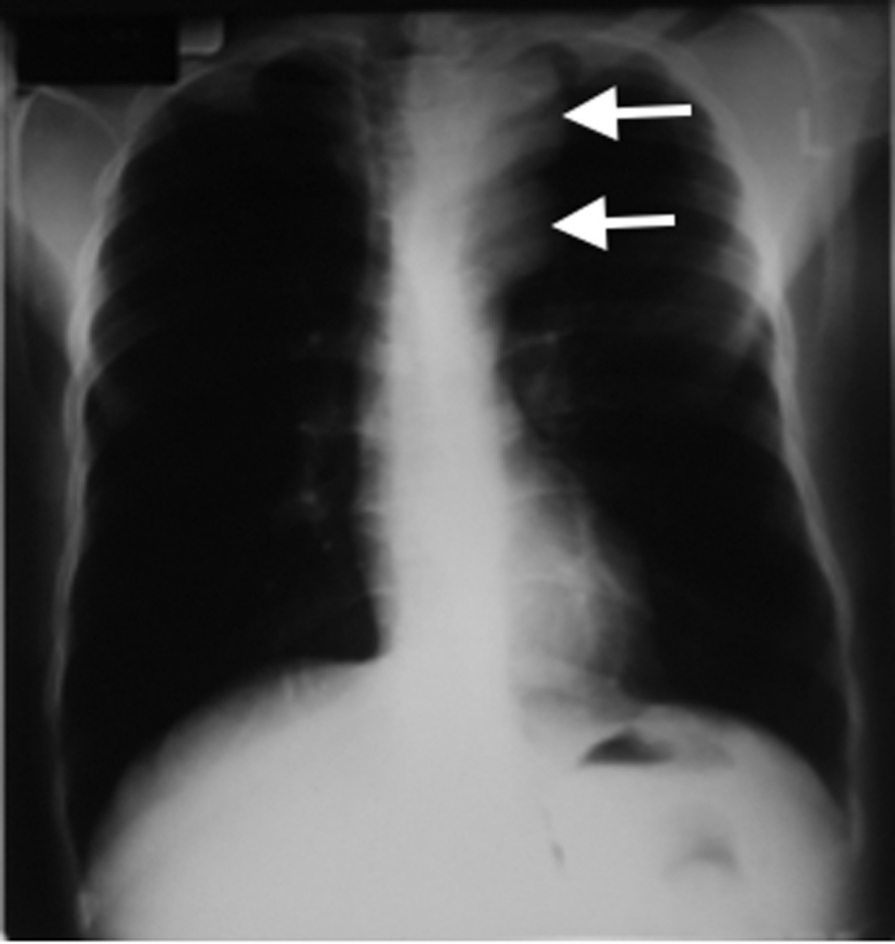
Chest radiograph showing superior mediastinal mass (arrows) and tracheal deviation.

Results of the ECG were normal. A Doppler ultrasound scan of the right neck mass showed a 6 cm × 6 cm pseudoaneurysm with a clot in the lumen. A chest radiograph showed a left paratracheal mass in the superior mediastinum causing tracheal deviation to the right. The lung fields were normal, and no other chest pathology was noted on the radiograph (Fig 1).

**Figure 2:**
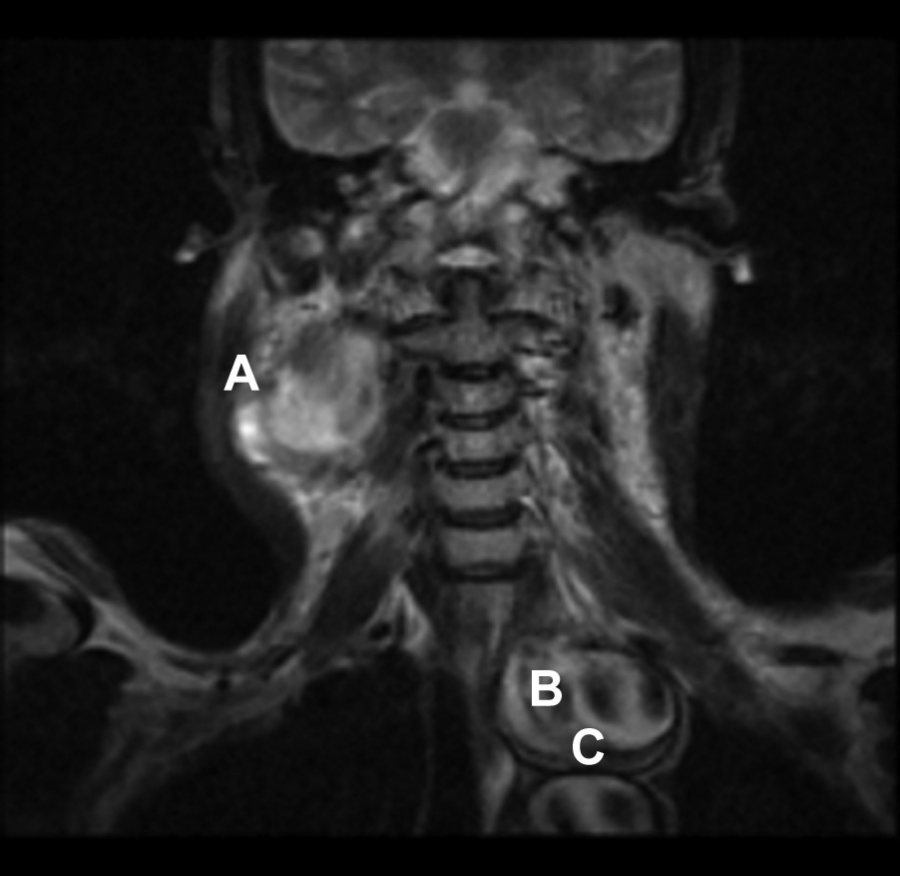
Noncontrast MRI scan of neck and upper thorax showing (A) right common carotid artery pseudoaneurysm, (B) proximal left subclavian artery pseudoaneurysm, and (C) pseudoaneurysm at the origin of the left subclavian artery.

A non-contrast MRI scan of the neck and upper thorax showed three large pseudoaneurysms. One was a right common carotid artery pseudoaneurysm, 6 cm in diameter, with extensive mural thrombus. Additionally, there were two intrathoracic pseudoaneurysms—a superior one, 6 cm wide, encircling the proximal left subclavian artery, and an inferior one, 4 cm wide, at the origin of the left subclavian artery (Fig 2).

*What is the diagnosis?*

*Diagnosis:* TB-/HIV-associated large vessel pseudoaneurysms

## Discussion

Pseudoaneurysms and the finding of multiple pseudoaneurysms at once have been reported in the context of both TB and HIV infections but their incidence and clinical progression in HIV and TB co-infected patients are unknown.

Tubercular pseudoaneurysms occur in both immunocompetent and immunosuppressed individuals. They are thought to arise from infection of blood vessels by *Mycobacterium tuberculosis*. Mycobacteria reach the blood vessel wall in four main ways: (a) direct implantation of bacilli on the vessel wall in miliary TB, (b) spread to the adventitia or media via the vasa vasorum, (c) spread to the vessel wall via lymphatics of the vasa vasorum, and (d) extension from a neighboring tuberculous lymph node, abscess, or bone. Thoracic pseudoaneurysms may occur in association with pulmonary TB. Tubercular pseudoaneurysms may, however, occur at other sites, such as the iliac, femoral, and popliteal arteries. The age at presentation varies widely from 13 to 60 years.

In contrast, the cause of HIV-associated pseudoaneurysms is incompletely understood. Proposed mechanisms include (a) direct vessel wall injury by HIV or opportunistic co-infections such as cytomegalovirus and Epstein-Barr virus; (b) indirect action through formation and deposition of immune complexes on vessel walls; (c) aberrant HIV-associated immune activation or necrotizing inflammation; and (d) immune reconstitution inflammatory syndrome and restoration of pathogen-specific cellular immune responses during ART. The anatomical sites commonly affected are the common carotid arteries, thoracic and abdominal aorta, and common iliac, femoral, and popliteal arteries. Several patterns of vasculitis, including granulomatous inflammation, leukocytoclastic vasculitis of the vasa vasorum, adventitial inflammation, and focal necrotizing vasculitis, have been reported, but atherosclerosis is typically absent. The disease has a predilection for young adult males, particularly in the fourth decade, and has been described predominantly in black Africans. Epidemiological studies indicate that HIV-associated pseudoaneurysms are rare in the era of effective ART, suggesting that the degree of HIV control impacts the development of HIV-associated vasculopathy.

The clinical presentation of pseudoaneurysms varies by anatomical site. Symptoms range from pain at the site of pseudoaneurysm, hoarseness, dysphagia, a palpable pulsatile mass, and evidence of acute deterioration from bleeding into the GI tract, lungs, peritoneal cavity, or pericardial space.

Investigations for pseudoaneurysms include a variety of radiological imaging techniques such as contrast-enhanced CT scan and MRI to visualize the lesions and surrounding organs. CT and magnetic resonance angiography delineate and enhance details of the vessels. Additional imaging techniques such as fluorodeoxyglucose PET and gallium scanning may be performed to establish the extent of vessel inflammation. For superficial aneurysms, Doppler ultrasound scanning may be used. Blood tests to determine the degree of inflammation such as erythrocyte sedimentation rate and C-reactive protein should be included in the workup of suspected infection-associated or inflammatory pseudoaneurysms. For suspected TB pseudoaneurysms, additional investigations for TB such as radiographs of the chest and other anatomical sites of disease, sputum microscopic examination for AFB, nucleic acid amplification tests (Xpert MTB/RIF and Xpert MTB/RIF Ultra), mycobacterial culture, urine lipoarabinomannan (for people living with HIV), and tissue sampling from the site of disease for histological examination should be considered. For patients with HIV-associated pseudoaneurysms, measurements of peripheral blood CD4 count and HIV viral load are recommended.

Treatment of pseudoaneurysms generally requires a combination of medical therapy and surgery. For tubercular pseudoaneurysms, anti-TB drug therapy alone may not be adequate, because there is a risk of persistent infection and rupture. Patients with HIV-associated pseudoaneurysms should receive effective ART, as would be recommended for other HIV-associated complication. Surgery should be performed urgently for both tubercular and HIV-associated pseudoaneurysms regardless of the size, because rupture may occur at any size. Surgical treatment may be open or endovascular, depending on the location and length of the pseudoaneurysm, age and general condition of the patient, and the surgeon’s experience. Endovascular surgery to insert a stent at the site of the pseudoaneurysm is minimally invasive and a preferred option for patients who cannot withstand open surgery. Although embolization has been used extensively to treat visceral or iatrogenic small vessel aneurysms, it has not been used to treat TB- or HIV-associated large vessel pseudoaneurysms. Recurrence in other sites after resection has been reported in both tubercular and HIV-associated pseudoaneurysms.

This case highlights the challenges of diagnosing and managing patients with pseudoaneurysm in resource-limited settings with high prevalence of both HIV and TB. Some blood and microbiology tests that should have been performed in the workup of this patient were unavailable at the hospital. MRI and CT imaging are not readily available, limiting the capacity to confirm the diagnosis radiologically. Despite the limited investigations, the attending clinical team managed the patient by applying good clinical judgment informed by knowledge of local disease patterns. Finally, vascular surgery is not often available, and most patients cannot afford to travel to other countries for vascular surgery. A combination of these factors inevitably contributes to the high mortality associated with pseudoaneurysms in low-resource settings.

### Clinical Course

He was started on ART comprising tenofovir, lamivudine, and efavirenz. Because of the lack of capacity for major vascular surgery in Malawi, the patient made private arrangements for surgery in South Africa. We were unable to follow his progress while in the hospital in South Africa or to access details of the care he received while there, but we were informed by family members that he initially had successful surgical repair of the right common carotid artery pseudoaneurysm but died of complications of surgery for intrathoracic pseudoaneurysms.

Although a definitive histological diagnosis of the pseudoaneurysms was not established, the history of sputum smear-positive TB strongly suggests TB as a likely cause. It is not possible to exclude HIV infection as a contributing or independent cause. Furthermore, the FNA of the enlarged right cervical lymph nodes may have promoted local spread of bacilli from the lymph nodes or may have caused trauma to the right common carotid artery, but the finding of other pseudoaneurysms in noncontiguous sites makes a systemic rather than local mechanism more likely.

## Clinical Pearls

1. *Pseudoaneurysms are a complication of both TB and HIV infection. Multiple mechanisms underlie their pathophysiology.*
2. *TB - and HIV-associated pseudoaneurysms can present at multiple sites including thoracic and abdominal aorta, iliac, femoral, and popliteal arteries.*
3. *Treatment of pseudoaneurysms comprises medical therapy depending on the cause, and surgical repair.*
4. *Investigation and management of pseudoaneurysms in low-resource settings is limited by the availability of appropriate resources and expertise. Good clinical judgment and knowledge of local disease patterns are essential.*

## References

[bib1] Calabrese L.H., Estes M., Yen-Lieberman B. (1989). Systemic vasculitis in association with human immunodeficiency virus infection. Arthritis Rheum.

[bib2] Nair R., Abdool-Carrim A., Chetty R., Robbs J. (1999). Arterial aneurysms in patients infected with human immunodeficiency virus: a distinct clinicopathology entity?. J Vasc Surg.

[bib3] Chello M. (2002). Pseudoaneurysm of the thoracic aorta in patients with human immunodeficiency virus infection. Eur J Cardio-Thoracic Surg.

[bib4] Heikkinen M.A., Dake M.D., Alsac J.-M., Zarins C.K. (2005). Multiple HIV-related aneurysms: open and endovascular treatment. J Endovasc Ther.

[bib5] Jain A.K., Chauhan R.S., Dhammi I.K., Maheshwari A.V., Ray R. (2007). Tubercular pseudoaneurysm of aorta: a rare association with vertebral tuberculosis. Spine J.

[bib6] Robbs J.V. (2009). Pathogenesis and pathology of HIV-related large-vessel disease. S Afr J Surg.

[bib7] Ishizaka N., Sohmiya K., Miyamura M. (2012). Infected aortic aneurysm and inflammatory aortic aneurysm: in search of an optimal differential diagnosis. J Cardiol.

[bib8] Ju-Mei C., Hassan L., Skinner G.C. (2017). Multiple large vessel aneurysmal formation in HIV-infected patients. SA J Radiol.

[bib9] Pallangyo P., Lyimo F., Bhalia S. (2017). Bilateral multiple pulmonary artery aneurysms associated with cavitary pulmonary tuberculosis: a case report. J Med Case Rep.

[bib10] Xue J., Yao Y., Liu L. (2018). Treatment of tuberculous aortic pseudoaneurysm associated with vertebral tuberculosis: a case series and a literature review. Medicine.

